# 18ï ¢-Glycyrrhetinic acid suppresses glioblastoma by regulating p38 signaling pathway: an integrative approach combining network analysis, transcriptomics, and experimental assessment

**DOI:** 10.3389/fphar.2026.1727072

**Published:** 2026-02-17

**Authors:** Xuanzhi Luo, Yuan Zeng, Xue Wang, Mujin Li, Ya-Lei Wang, Tiantian Peng, Cuiyan Ma, Qian Hua

**Affiliations:** 1 School of Life Sciences, School of Traditional Chinese Medicine, Beijing University of Chinese Medicine, Beijing, China; 2 Department of Pharmacy, Beijing Tsinghua Changgung Hospital, School of Clinical Medicine, Tsinghua University, Beijing, China; 3 University of Leeds, Leeds, United Kingdom; 4 Chinese Institutes for Medical Research, Beijing, China

**Keywords:** 18β-Glycyrrhetinic acid, glioblastoma, integrative bioinformatics, MAPK11, p38 signaling pathway

## Abstract

**Background:**

Glioblastoma (GBM) remains a therapeutic challenge with limited treatment options and poor prognosis. 18β-Glycyrrhetinic acid (GA), a natural metabolite from licorice, has shown anti-tumor potential, but its pharmacological effects in GBM and the underlying mechanisms require systematic investigation.

**Methods:**

The anti-GBM activity of GA *in vitro* was evaluated in GBM cells using CCK-8, colony formation, wound healing, and flow cytometry assays. Intracranial and subcutaneous GBM models in C57BL/6 and nude mice were established to assess the *in vivo* pharmacological effects of GA. An integrative approach combining network analysis, transcriptomic sequencing, and TCGA data analysis was employed to explore key genes and pathways. Molecular docking predicted GA binding to MAPK11, and Western blotting assessed its impact on p38 signaling pathway.

**Results:**

GA significantly inhibited GBM cell proliferation, migration, and induced apoptosis *in vitro*. *In vivo*, GA treatment markedly suppressed tumor growth in both intracranial and subcutaneous models, with no observed toxicity. Integrated bioinformatics analysis revealed that high MAPK11 (p38-pathway) expression was significantly associated with poor patient prognosis in TCGA. Molecular docking confirmed a strong binding affinity between GA and MAPK11. Mechanistically, GA downregulated MAPK11 expression, activated p38 signaling pathway, and subsequently suppressed the MEK/ERK signaling pathway.

**Conclusion:**

This study demonstrats that GA exerted potent anti-GBM effects by regulating p38 signaling pathway, provides novel mechanistic insights, and positions its as a promising therapeutic candidate against GBM.

## Graphical Abstract

**Figure:**
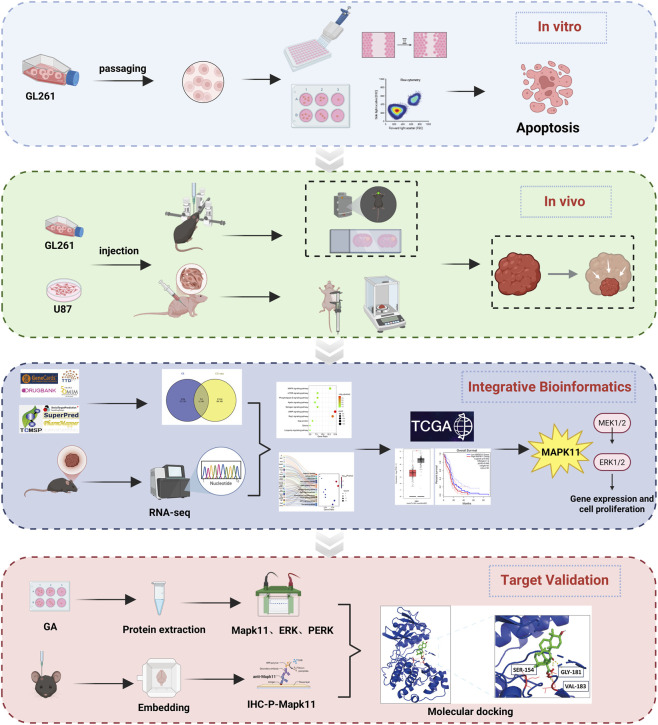

## 1 Introduction

Glioma is one of the most common primary intracranial tumors, originating from glial cells. It is leading cause of death from primary brain tumors (Lamba et al., 2021). Based on histological characteristics and the 2021 WHO classification of central nervous system (CNS) tumors, glioblastoma (GBM) is the most malignant form of glioma. The current standard treatment involves maximal safe surgical resection, followed by radiotherapy and adjuvant temozolomide (TMZ) chemotherapy (Stupp et al., 2005; Schaff and Mellinghoff, 2023). The invasiveness of GBM presents a major obstacle to achieving complete surgical resection (Giese et al., 2003). This makes postoperative adjuvant therapy essential for patient prognosis. However, about 60% of patients show a poor response to TMZ. Median survival following combined TMZ-based chemoradiotherapy ranges between 14 and 17 months (Pouyan et al., 2025; Lassman et al., 2023). Furthermore, TMZ is associated with toxic side effects and a high propensity for inducing drug resistance (Begagić et al., 2023). Although basic research on glioma has advanced in recent years, successful clinical translation remains a major bottleneck. The efficacy of novel therapies has been unsatisfactory, and GBM patients still face a poor overall prognosis.

Postoperative GBM readily develops drug resistance due to tumor heterogeneity (Zong et al., 2015). To address this challenge, multi-targeted natural products have emerged as a key source for anticancer drug development (Atanasov et al., 2021). Among these, active components from traditional Chinese medicine demonstrate significant potential. 18β-Glycyrrhetinic acid (GA), the main active metabolite of licorice, has been identified in previous studies to possess extensive pharmacological effects, including antioxidant (Han et al., 2024), anti-inflammatory (Ran et al., 2024), immunomodulatory (Li et al., 2022; Han et al., 2024), metabolic regulation (Yang et al., 2025a), and apoptosis induction activities (Zhang et al., 2022). GA has been shown to exert antitumor effects in various cancers, including hepatocellular carcinoma (Wang et al., 2020), gastric cancer (Yang et al., 2023), breast cancer (Wen et al., 2021), and non-small cell lung cancer (Guo et al., 2024). Its multifaceted mechanisms include inducing apoptosis, promoting ferroptosis, and activating autophagy.

Currently, research on GA-mediated inhibition of GBM remains limited. Only one study has reported that GA can suppress glioma cell proliferation, though its molecular mechanism is still underexplored and elusive (Genc et al., 2025). In this study, we demonstrated the inhibitory effect of GA on GBM both *in vitro* and *in vivo*. Furthermore, integrated bioinformatics and transcriptomics analyses-validated with the TCGA database-suggested that MAPK11 (p38-pathway) may interact with GA. Our findings showed that GA inhibited GBM cell proliferation and induced apoptosis by activing p38 signaling pathway.

## 2 Materials and methods

### 2.1 Cell culture and viability assay

GL261 (Jinyuan Biotechnology, Shanghai), U87 (Fuheng Biotechnology, Shanghai), and CT2A cells (obtained from Beijing Tiantan Hospital) were cultured in DMEM/F12, MEM, and DMEM medium. All media were supplemented with 10% fetal bovine serum (FBS; Life Technologies/Gibco, United States), 1% penicillin/streptomycin (Life Technologies/Gibco, United States), and cells were incubated at 37 °C in a humidified 5% CO_2_ atmosphere. Cell viability was evaluated using the Cell Counting Kit-8 (CCK-8) assay.

GL261 (5,000 cells/well), U87 (5,000 cells/well), and CT2A (7,000 cells/well) were seeded in 96-well plates. Cells were treated with a gradient concentration of GA (0, 20, 30, 40, 50, 60, 70, 80, 90, and 100 μM) for 24 h. Subsequently, CCK-8 reagent was added and incubated for 2 h. Absorbance was assessed at 450 nm.

### 2.2 Colony formation assay

GL261 cells were seeded in 6-well plates at a density of 400 cells per well. After 24 h, the cells were treated with 30, 50, and 70  μM GA and continuously cultured for 10 days. The culture medium was then removed, and the cells were washed with PBS, fixed with 4% paraformaldehyde for 10 min, and stained with 0.1% crystal violet (Solarbio, China) for 15 min. And the colonies were photographed and quantified using ImageJ.

### 2.3 Cell migration assay

GL261 cells were seeded in 6-well plates at a density of 1 × 10^5^ cells per well. After reaching 90% confluence, a uniform scratch was created using a sterile pipette tip, and the cells were washed twice with PBS. The cells were then treated with 30, 50, and 70 μM of GA Images of the wound area were captured at 0 and 24 h. The cell migration rate was calculated as follows: Migration rate (%) = [1 − (Wound area at 24 h/Wound area at 0 h)] × 100%.

### 2.4 Apoptosis analysis by flow cytometry

Apoptosis was evaluated using an Annexin V-FITC/propidium iodide (PI) Apoptosis Detection Kit (Beyotime, China). Cells were treated with GA (0 μM,30 μM,50 μM,70 μM) for 24 h, harvested, and stained according to the manufacturer’s protocol. Flow cytometry was performed using a Beckman CytoFLEX (Beckman Coulter Inc), and data were analyzed with FlowJo software.

### 2.5 GBM xenograft model

#### 2.5.1 Intracranial GBM model

Male C57BL/6 mice (6–8 weeks old, 20–22 g, SPF grade) were obtained from SPF (Beijing) Biotechnology Co., Ltd. (Beijing, China). GL261 cells (5 × 10^4^ cells/mouse) were stereotactically injected into the right brain (0.5 mm posterior, 2 mm lateral to the bregma, 2.5 mm deep). The needle was retained for 7 min before slow withdrawal, and the scalp was sutured. After 7 days, mice were randomly divided into five groups. Following 13 days of treatment, D-luciferin K^+^ salt (Aladdin) was injected, and tumor growth was monitored using the IVIS Spectrum imaging system.

#### 2.5.2 Subcutaneous GBM model

Male BALB/c nude mice (6–8 weeks old, 20–22 g, SPF grade) were obtained from SPF (Beijing) Biotechnology Co., Ltd. (Beijing, China). U87 cells (1 × 10^6^ cells/mouse) were subcutaneously injected. After 6 days, mice were divided into five groups. Tumor volume was measured every 2 days and calculated using the formula length × width^2^/2.

Both intracranial and subcutaneous models were treated daily via oral gavage with TMZ (35 mg/kg/day, Selleck) or GA (12.5, 25, or 50 mg/kg/day, Aladdin, Moligand™, ≥97%) for 13 days, while the control group received Carboxymethyl cellulose sodium salt solution (CMC-Na).

### 2.6 Network analysis

Potential targets of GA were predicted using the SuperPred, TSCMP, PharmMapper and SwissTargetPrediction databases, while GBM-related targets were collected from GeneCards, OMIM, DrugBank, TTD databases and literature (Darendelioglu et al., 2025; Ding et al., 2025; Gelen et al., 2025; Keshri et al., 2025; Li et al., 2025; Lu et al., 2025; Peng et al., 2025; Qin et al., 2026; Shanahati et al., 2025; Xu et al., 2025; Xue et al., 2025; Yang et al., 2025; Zhang et al., 2025; Zhu et al., 2025) through Pubmed. After standardization and removal of duplicates, the overlapping targets between GA and GBM were subjected to protein-protein interaction (PPI) network construction using the STRING database, followed by KEGG and GO enrichment analyses through the DAVID database.

### 2.7 Transcriptomic sequencing

cDNA libraries were sequenced on the Illumina platform by Novogene Co., Ltd. (Beijing, China). Tumor samples from the control group (treated with CMC-Na) and the high-dose GA group (50 mg/kg/day) were subjected to RNA extraction using TRIzol. RNA integrity was assessed using the Agilent 2,100 Bioanalyzer. cDNA synthesis and library preparation were performed by Novogene. After quality control, high-throughput sequencing was carried out on the Illumina platform.

### 2.8 Metabolite–target–pathway diagram

Based on the 10 proliferation- and apoptosis-related KEGG pathways, a “GA–disease target–pathway” network was constructed using Cytoscape, and key nodes were identified via topological analysis.

### 2.9 Key-gene analysis in GEPIA

The GEPIA database was used to analyze the correlation between the expression of 45 key genes (from the metabolite–target–pathway diagrams) and patient survival. Kaplan–Meier survival curves were generated using the “Survival Analysis” module with default parameters. 2.7 Immune infiltration analysis.

The TIMER database was used to evaluate the correlation between MAPK11 expression and immune cell infiltration levels. Using the “Immune Association” module, purity-corrected Spearman correlations were calculated between MAPK11 expression and infiltration of B cells, CD4^+^ T cells, CD8^+^ T cells, macrophages, neutrophils, and dendritic cells.

### 2.10 Western blotting

GL261 cells were treated with GA (0, 30, 50, 70 µM) for 24 h. Afterward, whole-cell extracts were prepared. 10% SDS-PAGE resolved proteins, transferred to PVDF membranes, and blocked with 5% skim milk. The membranes were then incubated overnight at 4 °C with primary antibodies against ERK (1:1,000, CST4695), p-ERK (1:1,000, CST4370), and MAPK11 (1:1,000, abcam ab32142), p-p38 (1:1,000, CST4511), p-MAPKAPK2 (1:1,000, CST3007) followed by incubation with HRP-conjugated secondary antibodies (1:5,000, Jackson ImmunoResearch). ImageJ software was used to analyze protein bands, and the expression levels were normalized against GAPDH or Tubulin.

### 2.11 Tissue sectioning and immunohistochemistry

Mice were euthanized and perfused transcardially with saline, followed by 4% paraformaldehyde (PFA). Brains were fixed in 4% PFA at 4 °C for 12 h, embedded in paraffin, and sectioned at 4 μm. Primary tumor, heart, liver, spleen, lung, and kidney sections were stained with hematoxylin and eosin (HE). Brain sections were immunostained with Ki67 (1:1,000, abcam16667). Tissues were observed and imaged using a Leica microscope.

### 2.12 Molecular docking

The 3D structure of MAPK11 was obtained from the RCSB PDB database. The 2D structure of 18β-glycyrrhetinic acid was retrieved from PubChem and converted using Open Babel. AutoDock was used to predict binding sites, and PyMOL was employed for visualization of binding conformations.

### 2.13 Statistical analysis

Data are presented as the mean ± standard error of the mean (SEM) from n = 3 independent biological replicates. The exact value of n for each experiment is provided in the corresponding figure legend. In this study, biological replicates are defined as experiments performed independently on different cell culture passages or preparations.

All statistical analyses were performed using GraphPad Prism 10.1.2. Prior to parametric testing, the assumptions of normality and homogeneity of variances were formally assessed. The normality of data distribution for each group was verified using the Shapiro-Wilk test. The homogeneity of variances across groups was confirmed using Brown-Forsythe’s test (for multiple groups) or an F-test (for two groups).

Specific statistical tests were applied as follows: for comparisons between two independent groups, a two-tailed unpaired Student’s t-test was used. If the data violated the assumption of normality, the non-parametric Mann-Whitney U test was employed. For comparisons among more than two independent groups (e.g., dose-response experiments with 0, 30, 50, 70 μM GA), a one-way analysis of variance (ANOVA) was conducted, followed by Dunnett’s post hoc test for multiple comparisons against a single control group.

Statistical significance is denoted as follows: *p < 0.05, **p < 0.01, ***p < 0.001, ****p < 0.0001.

## 3 Results

### 3.1 GA inhibited GBM cells *in vivo*

**FIGURE 1:**
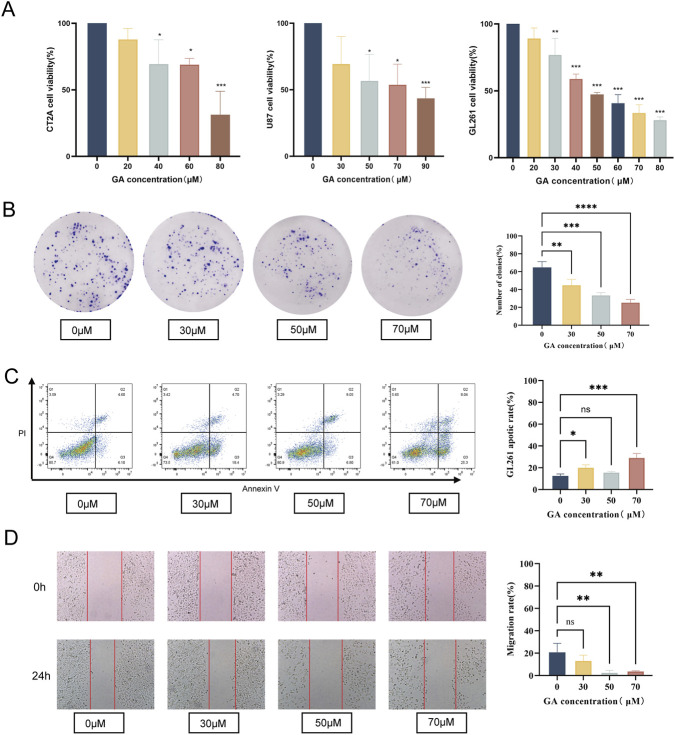
Effects of GA on proliferation, migration, and apoptosis in GBM cells. **(A)** U87, CT2A and GL261 were exposed to GA (0, 30, 50,70 μM) for 24 h. Cell viability was assessed using the CCK-8 assay (n = 3). **(B)** Colony formation assay of GL261 cells treated with GA (0, 30, 50, 70 μM) to evaluate clonogenic ability (n = 3). **(C)** Migration rates of GL261 cells treated with GA (0, 30, 50, 70 μM) were assessed using a wound healing assay (n = 3). **(D)** Flow cytometric analysis of the proportion of apoptotic GL261 cells after treatment with GA (0, 30, 50, 70 μM) (n = 3). Data are expressed as means ± SEM. Statistical significance was determined by one-way ANOVA. *p < 0.05, **p < 0.01, ***p < 0.001, ****p < 0.0001. GA: 18β-glycyrrhetinic acid.

To evaluate the effects of GA on GBM cells proliferation, three GBM cell lines (U87, CT2A, and GL261) were exposed to different concentrations of GA for 24 h. The findings demonstrated that GA significantly inhibited the viability of the 3 cell lines in a dose-dependent manner *in vitro* (Figure 1A).

The colony formation and migration assays results indicated that the colony formation and migration abilities of GL261 cells treated with GA were significantly lower than those of the control group (Figures 1B,C). Furthermore, flow cytometry confirmed significantly elevated apoptosis rates in GA-treated GL261 cells versus the controls (Figure 1D).

Collectively, these findings indicated that GA exerted anti-GBM activity *in vitro* by suppressing cell growth and migration and inducing apoptosis.

### 3.2 GA inhibited tumor growth *in vivo* in intracranial GBM mouse models

**FIGURE 2:**
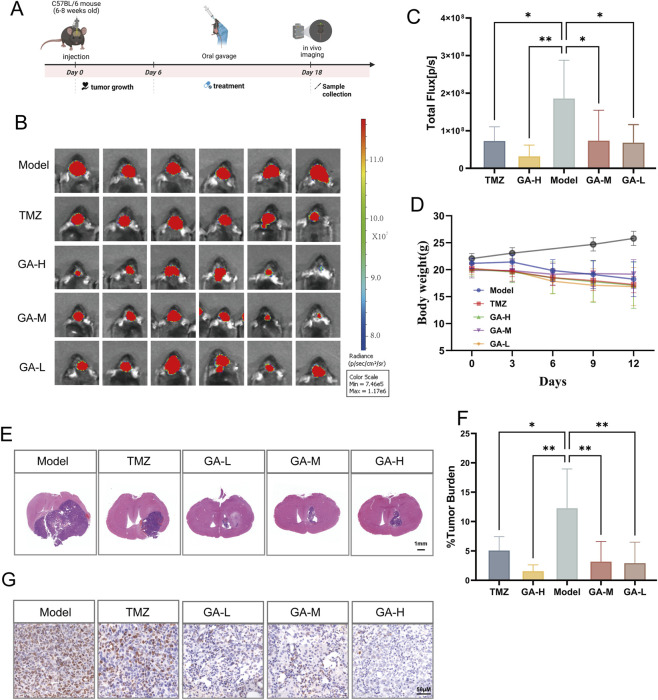
The inhibitory effects of GA in intracranial GBM models **(A)** The experimental timeline is schematically presented. **(B)** Bioluminescence images of the tumor-bearing mice on day 13 after GA or TMZ treatment, respectively (n = 6). **(C)** The quantitation of bioluminescence imaging was calculated (n = 6). **(D)** Body weights of the tumor-bearing mice after varied treatments (n = 6). **(E)** Representative histological images of HE staining (n = 5, scale bar:1 mm). **(F)** Percentage of tumor area relative to the brain section in HE staining (n = 5). **(G)** Representative images of IHC detection of Ki67 in the intracranial GBM model tissues across different groups (n = 3, scale bar: 50 μm). Data are expressed as means ± SEM. Statistical significance was determined by one-way ANOVA. *p < 0.05, **p < 0.01, ***p < 0.001, ****p < 0.0001. GA: 18β-Glycyrrhetinic acid. TMZ: Temozolomide. GA-H: Glycyrrhetinic acid high-dose group. GA-M: Glycyrrhetinic acid middle-dose group. GA-L: Glycyrrhetinic acid low-dose group. HE: Hematoxylin and eosin staining.

To clarify the effect of GA *in vivo*, we applied a GL261-luc intracranial GBM model, along with the treatment strategy outlined (Figure 2A). The tumor growth was significantly suppressed in the GA groups compared to the control group (Figures 2B,C), and no significant effect of GA on body weight was observed (Figure 2D). Histological analysis by HE staining confirmed a notable reduction in tumor size (Figures 2E,F), providing concrete evidence of its anti-GBM effect. To further evaluate the anticancer ability of the GA, tumor tissues of each group were stained with the immunohistochemistry staining of Ki67. There was a significant decrease in cancer cell proliferation compared with the control group (Figure 2G). Safety assessment *in vivo* indicated that GA was well-tolerated, with no observable toxicity or histopathological changes in major organs (Supplementary Material 1A,B).

### 3.3 GA inhibited tumor growth in subcutaneous GBM model

**FIGURE 3:**
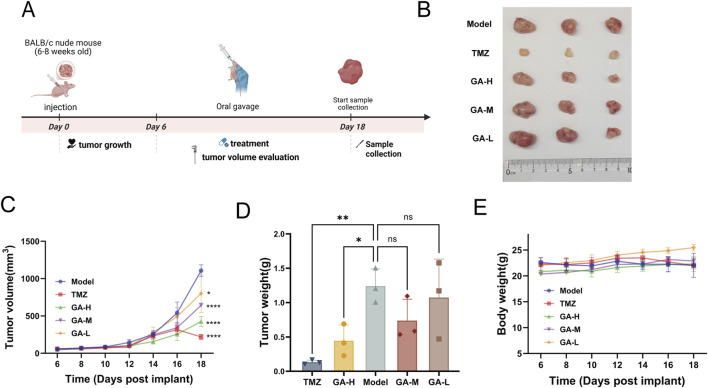
The inhibitory effects of GA in subcutaneous GBM models **(A)** The experimental timeline is schematically presented. **(B)** Tumor images of the subcutaneous tumor-bearing mice with different treatments (n = 3). **(C)** Tumor growth curve of subcutaneous tumors (n = 3). **(D)** The tumor weight of subcutaneous tumors (n = 3). **(E)** Body weights of the subcutaneous tumor-bearing mice after varied treatments. Data are expressed as means ± SEM. Statistical significance was determined by one-way ANOVA. *p < 0.05, **p < 0.01, ***p < 0.001, ****p < 0.0001. GA: 18β Glycyrrhetinic acid. GA-H: Glycyrrhetinic acid high-dose group. GA-M: Glycyrrhetinic acid middle-dose group. GA-L: Glycyrrhetinic acid low-dose group. HE: Hematoxylin and eosin staining.

We further established a subcutaneous GBM model to assess the inhibitory effect of GA, with the experimental timeline outlined in Figure 3A. Results showed that tumor volume was significantly reduced by medium- and high-dose GA and TMZ (Figure 3B). A pronounced decrease in tumor weight was also observed in the high-dose GA and TMZ groups (Figures 3C,D). In addition, no significant body weight changes were observed in the treatment relative to the control group, suggesting the excellent biosafety profile of the GA (Figure 3E).

### 3.4 MAPK11 may be a key target for the anti-GBM effects of GA

**FIGURE 4:**
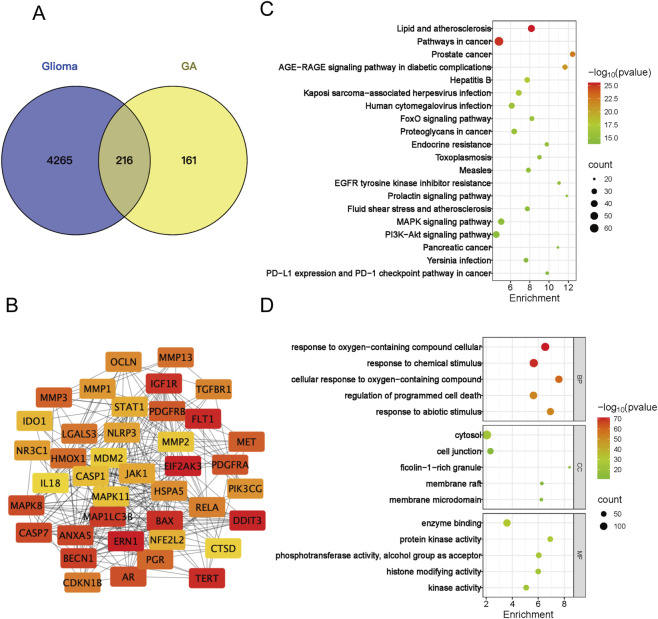
Network analysis -based analysis of GA’s functional impact. **(A)** Venn diagram of the overlapping targets between GA and GBM. **(B)** Protein-protein interaction (PPI) network of the top 40 core targets from the overlapping set in **(A,C)** Top 20 tumor-associated pathways from the KEGG enrichment analysis. **(D)** Top five significantly enriched Gene Ontology (GO) terms for the overlapping targets in **(A)** BP, biological process; CC, cellular component; MF, molecular function. GA:18β-Glycyrrhetinic acid.

Potential targets of GA were retrieved and predicted using the SuperPred, TSCMP, Pharmmapper, and SwissTarget Prediction databases. Following standardization of gene nomenclature and duplicate removal, 377 potential targets were identified. GBM targets were collected from the GeneCards, OMIM, DrugBank, and TTD databases. After the same standardization and deduplication procedures, 4481 GBM targets were obtained. A Venn diagram identified 216 overlapping targets between these two sets (Figure 4A). These overlapping targets were imported into the STRING database to construct a protein-protein interaction (PPI) network. Using the CytoNCA plugin, the top 40 hub targets were screened out, and their interaction network is presented in Figure 4B. For Go and KEGG pathway analysis, the top 20 tumor-associated signaling pathways are shown and the top 5 GO terms in Figures 4C,D.

**FIGURE 5:**
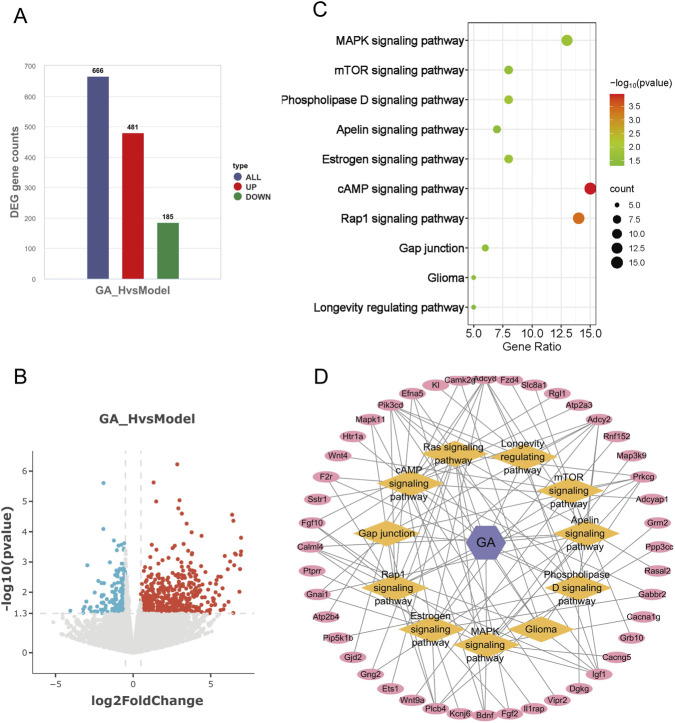
Integrative multi-omics identified GA’s functional impact. **(A)** Number of upregulated and downregulated genes in high-dose GA treatment groups. **(B)** Volcano plot of differentially expressed genes between the high-dose GA group and the model group. **(C)** Eleven overlapping pathways related to cell proliferation and apoptosis identified by integrating network analysis and transcriptomics. **(D)** Metabolite–target–pathway network of GA and GBM. GA:18β-Glycyrrhetinic acid.

To elucidate the mechanism underlying GA-mediated inhibition of GBM, we performed gene expression profiling by RNA sequencing (RNA-seq). In the intracranial model, treatment with high-dose GA led to significant upregulation of 481 genes and downregulation of 185 genes (P < 0.05) (Figures 5A,B). KEGG pathway analysis of these differentially expressed genes revealed that the top 25 enriched pathways were primarily associated with neuron–GBM interactions, the immune microenvironment, and GBM apoptosis/proliferation. Consistent with previous findings confirming that GBM suppressed proliferation and promoted apoptosis in GBM, we intersected transcriptomic and network analysis data with proliferation- and apoptosis-related KEGG pathways, identifying 10 overlapping pathways (Figure 5C). Based on these pathways and genes, a metabolite–target–pathway diagram was constructed to depict the relationships between 45 key genes and 10 signaling pathways (Figure 5D).

**FIGURE 6:**
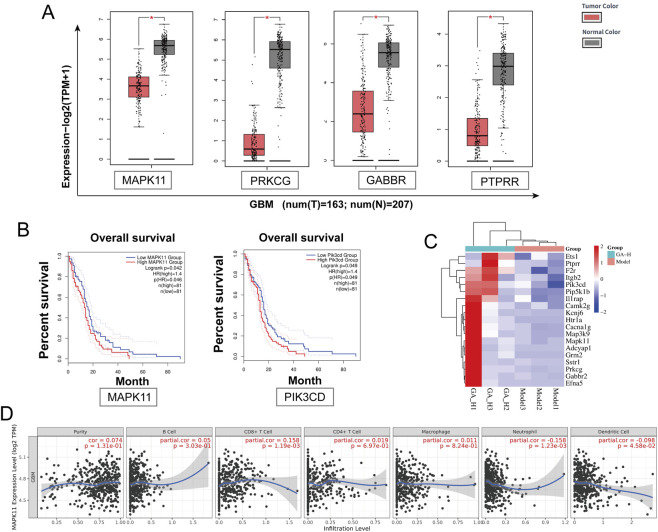
TCGA-based analysis identified MAPK11 as a key target of GA against GBM. **(A)** GEPIA-based comparison of MAPK11, PRKCG, GRB2, and PTPRR expression in GBM tumor tissues versus normal brain tissues. **(B)** Correlation between MAPK11 and PIK3CD expression levels and overall survival in GBM patients. **(C)** Heatmap of 19 key genes. **(D)** Association between MAPK11 expression and immune cell infiltration levels analyzed via TIMER. GA: 18β- Glycyrrhetinic acid.

We further examined the expression patterns of these 45 key genes in GBM and their potential links to the tumor microenvironment (TME). We analyzed the GEPIA database and identified 18 genes that were differentially expressed in glioma patients relative to healthy controls. Among these 18 genes, we highlighted that MAPK11 (Roche et al., 2020), PRKCG (Yang et al., 2025b), GBBR2 (Vinel et al., 2025), and PTPRR (Chang et al., 2023) are differentially expressed and have been linked to tumor development, which merits further study (Figure 6A). Of the 45 key genes, MAPK11 and PIK3CD expression levels were significantly linked to poorer patient outcomes (Figure 6B). We identified 19 genes from the GEPIA database that were either differentially expressed (18 genes) or associated with overall survival (2 genes), as shown in the heatmap (Figure 6C). Among the 19 genes in the heatmap, MAPK11 was the only one that was both differentially expressed and significantly prognostic, suggesting its potentially critical role in GBM development. Analysis of the TIMER database revealed a significant correlation between MAPK11 expression and the infiltration of multiple immune cell types, such as CD8^+^ T cells, dendritic cells, and neutrophils within the tumor microenvironment (Figure 6D).

### 3.5 GA exerted anti GBM effects by coordinated inhibition of the MEK/ERK and activation of the p38 pathways

**FIGURE 7:**
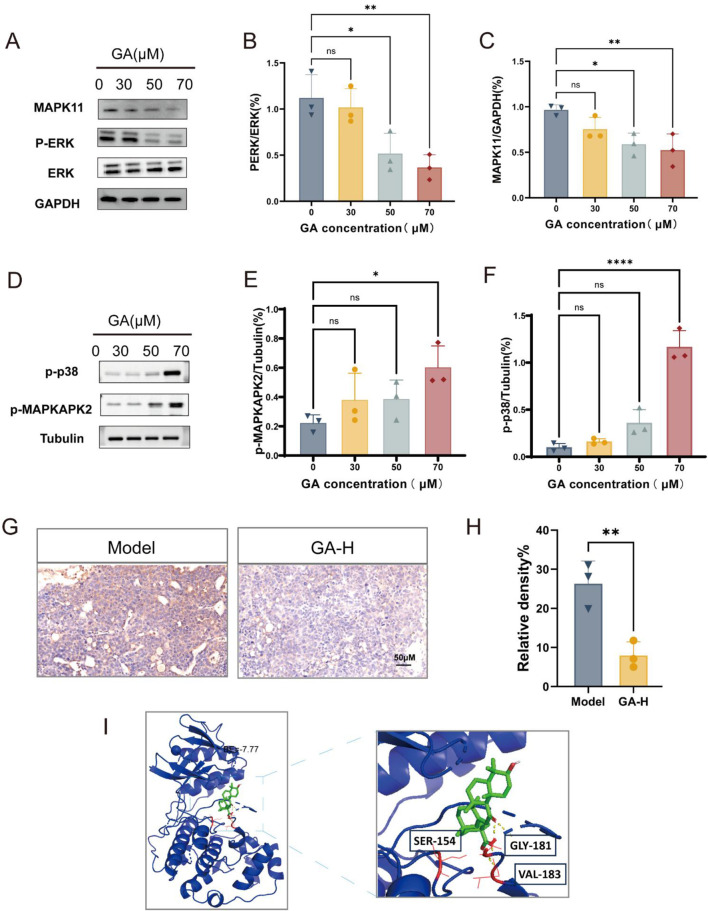
GA exerted anti-GBM effects by targeting MAPK11 and inhibiting MEK/ERK Pathway **(A–F)** Expression levels of MAPK11, total ERK, p-ERK, p-p38 and p-MAPKAPK2 in GL261 cells treated with GA (n = 3). **(G)** MAPK11 expression in GBM tissues from intracranial mice after GA treatment (n = 3, bar scale 50 μM). **(H)** The intensity of MAPK11 staining was quantified (n = 3). **(I)** Three-dimensional representation of the binding mode between GA and MAPK11. Data are presented as mean ± SEM. Statistical significance was determined by one-way ANOVA with Dunnett’s test for multiple-group comparisons **(A–F)** or by an unpaired Student’s t-test for two-group comparison **(H)** *p < 0.05, **p < 0.01, ***p < 0.001, **p < 0.0001. GA: 18β- Glycyrrhetinic acid. GA-H: Glycyrrhetinic acid high-dose group. p-ERK: phosphorylated ERK.

Our results showed that GA downregulated the expression of MAPK11 and phosphorylation levels of ERK in GL261 cells in a dose-dependent manner, without significantly affecting total ERK levels (Figures 7A–C). Additionally, GA treatment increased the phosphorylation of p38 and its downstream substrate MAPKAPK2 (Figures 7D–F). In the intracranial model, high-dose GA (50 mg/kg) also markedly reduced MAPK11 expression in GBM tissues (Figures 7G,H). Molecular docking further supported MAPK11 as a direct target, showing a strong predicted binding affinity (binding energy: −7.77 kcal/mol) between GA and MAPK11 (Figure 7I). Taken together, these data suggested that the anti-GBM effects of GA involved suppression of the MEK/ERK pathway and activation of the p38 pathway. The observed downregulation of MAPK11, associated with the predicted binding of GA from docking studies, positions it as a candidate target worthy of further investigation.

## 4 Discussion

GBM is a highly aggressive malignant tumor with limited treatment options and a poor prognosis. In this study, we found that GA exhibited significant anti-GBM activity. The CCK-8 assay showed that GA inhibited cell viability in three GBM cell lines *in vitro* (Figure 1A). Colony formation, wound healing, and flow cytometry assays demonstrated that GA suppressed proliferation and migration and promoted apoptosis in GBM cells (Figures 1B–D). Furthermore, we established intracranial models and subcutaneous models. In the intracranial models, anti-tumor pharmacological effects were evaluated by bioluminescent images (Figure 2B) and representative HE staining (Figure 2E), while in subcutaneous models, tumor volume (Figure 3C) and tumor weight (Figure 3D) were measured. Results from both models consistently indicated that GA treatment markedly inhibited GBM growth. In anti-cancer drug development, safety is widely considered a paramount criterion. In our study, GA showed no significant toxicity in intracranial models, as evidenced by histological and functional analyses of major organs and renal/hepatic parameters (Supplementary Figure S1).

Our initial experiments confirmed that GA inhibits proliferation and promotes apoptosis in GBM. To systematically elucidate its anti-GBM mechanism, we integrated network analysis with transcriptomic analysis. Potential targets of GA were retrieved from the following databases: SuperPred (probability ≥51%, 109 targets), PharmMapper (normalized fit ≥0.5, 139 targets), SwissTarget (probability >0, 89 targets), TCMSP (6 targets), and PubMed (109 targets from recent literature up to 2025). After merging and removing duplicates, 377 unique GA-related targets were obtained. Glioma-related targets were collected from DrugBank (18 targets), OMIM (150 targets), GeneCards (Relevance score >1.2, 4,481 targets), and TTD (41 targets), resulting in 4,481 disease-related targets after deduplication. The intersection of these two sets was taken to identify 216 potential anti-GBM targets of GA (Figure 4A). The PPI network depicted in Figure 4B was constructed using the top 40 potential targets identified by the DMNC algorithm of the CytoNCA plugin. Subsequently, KEGG pathway enrichment analysis was performed to uncover the signaling pathways underlying the therapeutic effects of GA (Figure 4C).

Meanwhile, RNA sequencing revealed differentially expressed genes after high-dose GA treatment (Figures 5A,B) and their associated KEGG pathways. By cross-referencing these two sets of KEGG pathways, we identified 10 core pathways related to tumor proliferation and apoptosis (Figure 5C). Metabolite–target–pathway diagrams were generated based on these 10 pathways and the involved genes (Figure 5D), visually representing the 45 genes associated with these core pathways.

We focused on 45 genes associated with these pathways which were derived from RNA-seq differential expression analysis. Their expression and survival impact were assessed in the TCGA cohort (Figures 6A,B). From this analysis, Compared with healthy controls, MAPK11 expression was significantly altered in GBM patients and was strongly associated with overall survival. MAPK11 emerged as a potential candidate, as it was overlap between these 45 genes and the top 40 hub genes from PPI network (Figure 4B). Although TCGA data indicated overall low expression of MAPK11 in GBM patients, those with high MAPK11 expression exhibited significantly poorer prognosis, suggesting a potential tumor-promoting role.

We confirmed that GA downregulated MAPK11 expression both *in vivo* (Figures 7G,H) and *in vitro* (Figures 7A,C). We further examined ERK, MAPK11 and MAPKAPK2 phosphorylation levels by Western blotting. The results showed that GA dose-dependently suppressed ERK phosphorylation in a dose-dependent manner. In contrast, a contrasting activation of the p38 pathway was observed, as evidenced by upregulated phosphorylation of both p38 and MAPKAPK2 (Figures 7A–F). The molecular docking results suggested a potentially strong interaction between GA and MAPK11 (Figure 7I).

The p38 MAPK signaling pathway plays a critical regulatory role in tumorigenesis and progression, with its activation generally associated with the inhibition of cell proliferation and the promotion of apoptosis (Wagner and Nebreda, 2009). In glioma, activation of the p38 pathway effectively suppresses tumor cell growth and induces apoptosis (Yuan et al., 2019). This mechanism has also been confirmed in other cancers, such as hepatocellular carcinoma (Chiu et al., 2010) and lung cancer (Wu et al., 2022), indicating that the p38 pathway serves as a key suppressor of proliferation across multiple tumor types. Our study found that GA promotes the activation of the p38 pathway, suggesting that the anti-tumor effects of GA may also be achieved through activation of this pathway.

MAPK11 functioned as an oncogene implicated in multiple cancer types (Roche et al., 2020). It was upregulated in hepatocellular carcinoma (HCC), where ETV4 promotes hepatocarcinogenesis via MAPK11 (Qi et al., 2023). Additionally, MAPK11 was identified as a target of miR-516a-5p, which is sponged by circ-0001955, thereby facilitating HCC tumorigenesis (Yao et al., 2019). In breast cancer cells, MAPK11 was also highly expressed and activated through recruitment by TSPAN8, supporting cancer stemness maintenance and growth, ultimately contributing to chemotherapy resistance and disease progression (Fan et al., 2024). In lung cancer, MAPK11 participated in the p38 MAPK signaling pathway, and p38-specific inhibitors reduced cell viability and enhanced cisplatin sensitivity (Planchard et al., 2012). MAPK11 also promoted the initiation, invasion, and progression of prostate cancer (PAN et al., 2023). These findings underscore the role of MAPK11 as an oncogene across diverse tumors.

Furthermore, Subir et al. reported that GA effectively inhibited melanoma proliferation and enhanced immune responses (Juin et al., 2020). Analysis via the TIMER database revealed a significant correlation between MAPK11 expression and infiltration levels of various immune cells (Figure 6D). This led us to hypothesize that GA may exert effects not only on GBM cells but also on the tumor immune microenvironment. Together, these results suggest that GA is a potentially effective and safe therapeutic candidate for GBM.

Collectively, our study demonstrated that GA inhibited GBM proliferation and promoted apoptosis by regulating p38 signaling pathway. These findings provided a theoretical foundation for developing licorice-derived active metabolites as novel anti-tumor drug candidates.

## 5 Conclusion

In summary, our findings indicated that GA likely suppresses GBM growth and promotes apoptosis by regulating p38 signaling pathway. Moreover, GA exhibited no detectable toxicity *in vivo*. Its favorable safety profile, combined with significant anti-tumor pharmacological effects, highlights GA as a highly promising therapeutic candidate for GBM with substantial clinical translational potential.

## Data Availability

The data presented in the study are deposited in the NCBI (SRA) repository, accession number PRJNA1414150.
