# Supplementary material for: 18ï ¢-Glycyrrhetinic acid suppresses glioblastoma by regulating p38 signaling pathway: an integrative approach combining network analysis, transcriptomics, and experimental assessment
Source: Front Pharmacol. 2026 Feb 17;17:1727072. doi: 10.3389/fphar.2026.1727072 (PMC12953517; doi:10.3389/fphar.2026.1727072)
Supplement: Supplementary file 1 [file Table1.docx]

Supplementary Material

# Supplementary Data

## Supplementary Figures


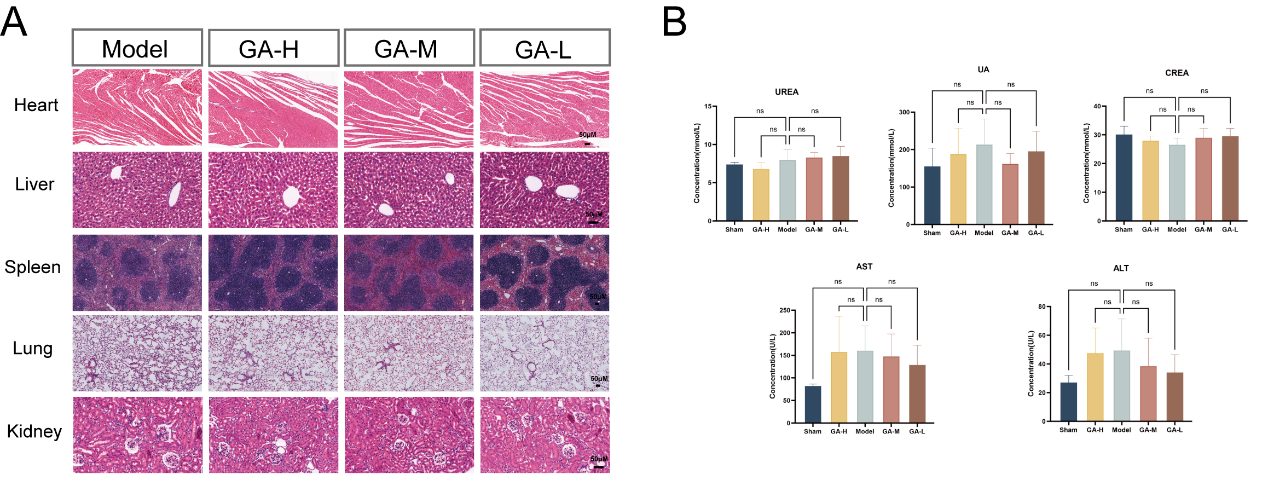


**Supplementary Figure 1.** **GA exhibited no obvious toxicity effects in vivo.** A. HE staining of heart, liver, spleen, lung, and kidney tissues from intracranial models treated for 13 days. B. Serum renal and hepatic function marker assays in intracranial models treated for 13 days.

GA: 18β-Glycyrrhetinic acid. GA-H: Glycyrrhetinic acid high-dose group. GA-M: Glycyrrhetinic acid middle-dose group. GA-L: Glycyrrhetinic acid low-dose group. HE: Hematoxylin and eosin staining.

1.2
