# Supplementary figures and images for: 18ï ¢-Glycyrrhetinic acid suppresses glioblastoma by regulating p38 signaling pathway: an integrative approach combining network analysis, transcriptomics, and experimental assessment
Source: Front Pharmacol. 2026 Feb 17;17:1727072. doi: 10.3389/fphar.2026.1727072 (PMC12953517; doi:10.3389/fphar.2026.1727072)

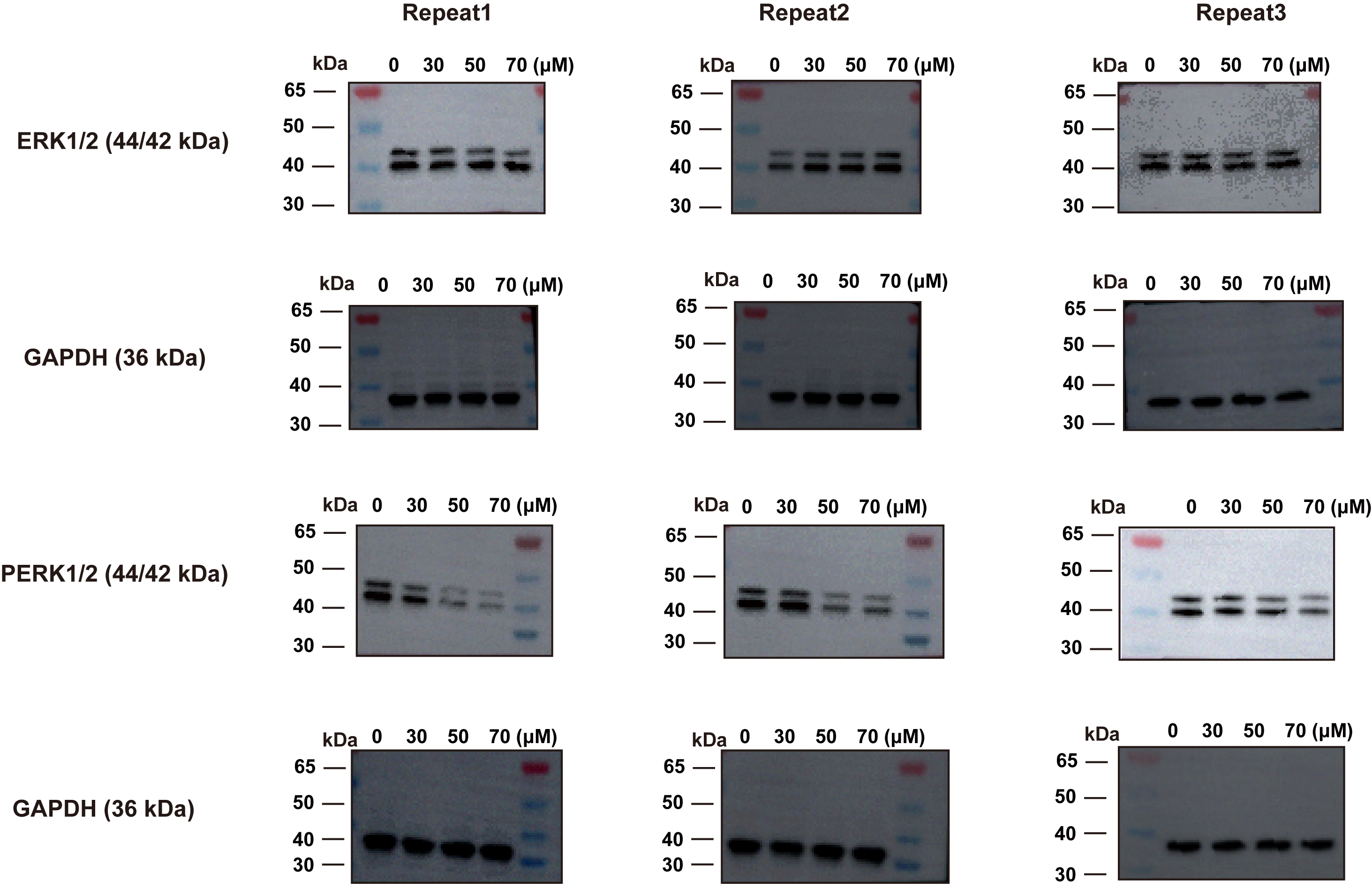

Supplement: Supplementary file 2 [file DataSheet1.zip › Western blotting/ERK_PERK_wb_merge.tif]

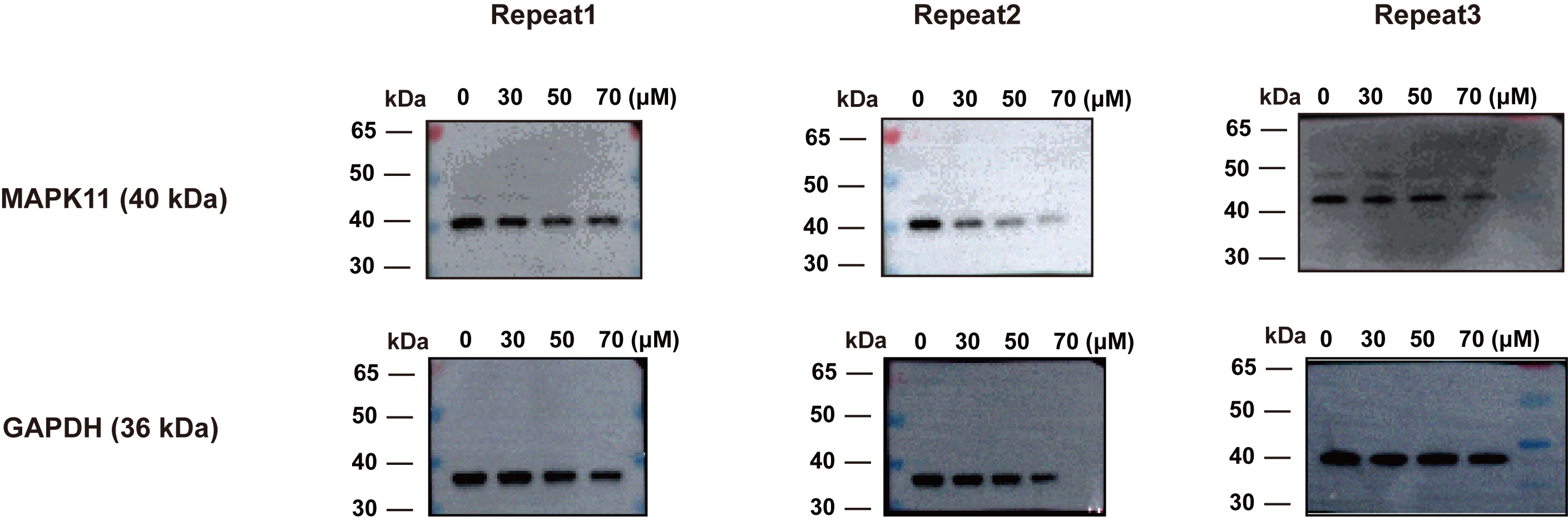

Supplement: Supplementary file 2 [file DataSheet1.zip › Western blotting/MAPK11_wb_merge.tif]

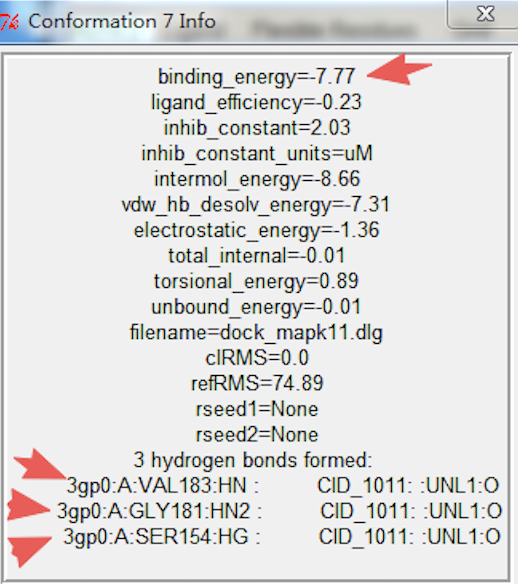

Supplement: Supplementary file 3 [file DataSheet2.zip › conformation info.jpg]
